# Doxycycline Inhibits IL-17-Stimulated MMP-9 Expression by Downregulating ERK1/2 Activation: Implications in Myogenic Differentiation

**DOI:** 10.1155/2016/2939658

**Published:** 2016-11-30

**Authors:** Hristina Obradović, Jelena Krstić, Tamara Kukolj, Drenka Trivanović, Ivana Okić Đorđević, Slavko Mojsilović, Aleksandra Jauković, Gordana Jovčić, Diana Bugarski, Juan Francisco Santibañez

**Affiliations:** Laboratory for Experimental Hematology and Stem Cells, Institute for Medical Research, University of Belgrade, Dr. Subotića 4, 11129 Belgrade, Serbia

## Abstract

Interleukin 17 (IL-17) is a cytokine with pleiotropic effects associated with several inflammatory diseases. Although elevated levels of IL-17 have been described in inflammatory myopathies, its role in muscle remodeling and regeneration is still unknown. Excessive extracellular matrix degradation in skeletal muscle is an important pathological consequence of many diseases involving muscle wasting. In this study, the role of IL-17 on the expression of matrix metalloproteinase- (MMP-) 9 in myoblast cells was investigated. The expression of MMP-9 after IL-17 treatment was analyzed in mouse myoblasts C2C12 cell line. The increase in MMP-9 production by IL-17 was concomitant with its capacity to inhibit myogenic differentiation of C2C12 cells. Doxycycline (Doxy) treatment protected the myogenic capacity of myoblasts from IL-17 inhibition and, moreover, increased myotubes hypertrophy. Doxy blocked the capacity of IL-17 to stimulate MMP-9 production by regulating IL-17-induced ERK1/2 MAPK activation. Our results imply that MMP-9 mediates IL-17's capacity to inhibit myoblast differentiation during inflammatory diseases and indicate that Doxy can modulate myoblast response to inflammatory induction by IL-17.

## 1. Introduction

Interleukin 17 (IL-17) family of cytokines has been intensively investigated in recent years. As a proinflammatory cytokine, IL-17 is involved in immune defense mechanisms acting through induction of secondary mediators of inflammation. However, its implication in various human inflammatory diseases such as rheumatoid arthritis and myositis was also demonstrated [1–3].

A number of data confirm the pleiotropic nature of IL-17. Its receptor (IL-17R) is ubiquitously expressed, in almost all cell types, allowing the widespread influence of IL-17 on cellular processes, through subsequent activation of numerous signal transduction pathways, such as protein kinase A, JAK/STAT, NF-*κ*B, and MAPKs, including ERK1/2 and p38 cascades [4, 5].

Proinflammatory cytokines are considered important mediators of skeletal muscle loss in various chronic diseases [6]. Known to be associated with the development of inflammatory diseases, IL-17 has also been implicated in myopathies, such as polymyositis and dermatomyositis [7, 8]. However, its role in muscle repair or regeneration has not been clarified yet. Skeletal muscle repair depends on satellite cells present* in situ*. These cells have the capability to proliferate, migrate to sites of injury, and differentiate into adult muscle cells [9]. During this process, the extracellular matrix (ECM) is dynamically and finely remodeled, while the imbalance in ECM reorganization can be involved in the development of muscle diseases [10]. Matrix metalloproteinases (MMPs) are a family of enzymes which can selectively digest individual components of the ECM [10, 11]. Several MMPs have been shown to be implicated in muscle regeneration, including matrix metalloproteinase type 2 (MMP-2) and matrix metalloproteinase type 9 (MMP-9). In skeletal muscle, MMP-2 is constitutively expressed, whereas MMP-9 shows none to minimal basal expression [10, 11]. In particular, MMP-9 expression and/or activity are tightly transcriptionally regulated and highly inducible in response to variety of agents, including proinflammatory cytokines [12]. In addition, increased expression and activation of MMP-9 are associated with various myopathies and inflammation-induced changes in skeletal muscle [10]. Moreover, the upregulation of MMP-9 expression has been described in the muscles of mdx mice, an animal model of Duchenne muscular dystrophy [13]. Even though high expression of both IL-17 and MMP-9 has been reported in inflammatory myopathies, the mutual relation of these proteins in muscle is still not well understood.

Doxycycline (Doxy) is an antibiotic of the tetracycline family of drugs and has been tested in numerous conditions associated with elevated MMP activity [14]. It has recently been demonstrated that Doxy can be beneficial for mdx mice mainly by reducing MMP-9 and TNF-*α* expression [15]. It was also demonstrated that Doxy or anti-MMP-9 antibody improves soleus muscle regeneration and ameliorate development of excessive fibrosis [16]. In addition, recent work showed that Doxy treatment can regulate both local and systemic inflammation, suggesting its importance in inflammatory myopathies [17, 18]. We have recently demonstrated the ability of IL-17 to inhibit myogenesis* in vitro* [19], while MMP-9 participation as well as the regulatory role of Doxy in this process has not been well defined so far.

Our findings indicate that IL-17 increases MMP-9 expression, along with the inhibition of C2C12 myoblast differentiation. Furthermore, results presented here show that Doxy protects C2C12 myoblasts from the capacity of IL-17 to inhibit myogenesis.

## 2. Material and Methods

### 2.1. Cell Culture

Myoblast C2C12 cell line was purchased from American Type Culture Collection (ATCC, Rockville, MD). Cells were cultured in growth medium (GM) consisting of Dulbecco's Modified Eagle's Medium (DMEM) supplemented with 10% Fetal Bovine Serum (FBS) from Capricorn Scientific (Ebsdorfergrund, Germany) and 100 Units/mL Penicillin and 0.1 mg/mL Streptomycin (Capricorn Scientific) in a humidified environment at 37°C and 5% CO_2_ in air. Myogenic differentiation was induced by culturing confluent cells in myogenic differentiation medium (MDM) consisting of GM supplemented with 2% horse serum instead of 10% FBS.

Recombinant mouse IL-17 was provided by R&D Systems (Minneapolis, MN, USA). Doxycycline was from Sigma-Aldrich (St. Louis, MO, USA). MEK1,2 inhibitor PD98059 was obtained from Calbiochem (Darmstadt, Germany) and used at 25 *μ*M.

### 2.2. Zymography Assay

MMP-9 and MMP-2 activities were examined as described previously [20]. Briefly, 50.000 cells/well were seeded in 24-well plates and cultured overnight; then, the cells were washed three times with PBS and 0.5 mL of serum-free culture medium was added. Cells were subsequently cultured with or without IL-17 as indicated for each experiment. During the induction of myogenic differentiation, cells were cultured for three days in MDM and additional 24 h to generate conditioned serum-free medium for MMP-9 determination. Protein concentration in conditioned media was determined by BCA Protein Assay Macro Kit (Serva, Heidelberg, Germany) according to manufacturer's instructions. Aliquots of protein normalized conditioned media were subjected to 8% SDS-PAGE containing 0.1% gelatin under nonreducing conditions. Gels were washed twice with 2.5% Triton X-100 and rinsed one time with distilled H_2_O. The gels were then incubated for 24 hours in 100 mM Tris-HCl, pH 8.5, with 10 mM CaCl_2_. The activity of MMPs was stopped by staining the gels with Coomassie Blue R250 in 50% methanol and 10% acetic acid for 20 minutes. Zymography was developed by destaining gels in 20% methanol and 5% acetic acid until transparent bands were visualized. Quantification of these areas was performed by densitometry analysis using NIH-Image J software.

### 2.3. Myotube Hypertrophy Index

C2C12 cells were grown in 24-well plates until reaching 90% confluence and then subjected to myogenic differentiation by replacing GM with MDM. Cells were cultivated for 4 days with indicated treatments. Cell monolayers were washed twice with PBS and fixed with ice cold methanol for 2 min, then stained with crystal violet (0.1%) for 15 min, and extensively washed with tap water. This strongly stained myotubes and nuclei within. To determine the hypertrophy index, myotube diameter was determined according to Yeh et al. [21]. Cultured cells were photographed using 10x objective lens and NIH-Image J software was used to measure myotube diameter. At least, 50 myotubes were used to determine the diameters and 3 short-axis measurements were taken along the length of a given myotube diameter and the average was calculated.

### 2.4. Western Blot Assay

Protein expression was analyzed by Western blot assay. Antibodies against myosin heavy chain (MyHC), phospho-ERK1/2 (sc-7383), and ERK1 (sc-94) were purchased from Santa Cruz Biotechnology (Santa Cruz, CA, USA). Mouse monoclonal anti-HA antibody was kindly provided by Dr. Carmelo Bernabeu (CIB, Spain). Cells were lysed in lysis buffer (50 mM Tris-HCl, pH 7.5, 150 mM NaCl, 1% NP-40) containing 2 mM EDTA, 50 mM NaF, and protease inhibitor cocktail (SERVA Electrophoresis GmbH, Heidelberg, Germany). Equal amounts of protein from each sample were separated by SDS-PAGE and transferred to nitrocellulose membranes (AppliChem, Darmstadt, Germany). Membranes were blocked with 4% BSA in 0.5% Tween-20 in TBS and then incubated with primary antibodies. Membranes were then incubated with secondary antibodies conjugated with HRP (Sigma-Aldrich). Labeled proteins were visualized using enhanced chemiluminescence reagent system from AppliChem. Protein bands were quantified by densitometry scanning, using NIH-Image J software and expressed relative to tubulin or corresponding total protein signals.

### 2.5. RT-PCR

Two micrograms of total RNA isolated from C2C12 cells was reverse transcribed using Superscript II (Invitrogen). PCRs were performed using One-Step PCR (Invitrogen) with the following settings: 94°C for 5 min, 26–30 cycles at 94°C for 45 sec, 52–54°C for 30 sec, and 72°C for 90 sec. The primer sets, annealing temperatures, number of cycles, and product size for each gene are given in Supplementary Table  1 (in Supplementary Material available online at http://dx.doi.org/10.1155/2016/2939658) [22, 23]. Amplicons were resolved in 1.5% agarose gel stained with ethidium bromide. The intensity of the bands was quantified using NIH-Image J software.

### 2.6. Plasmids, Transient Transfection, and Reporter Assays

The reporter construct pMMP-9-luc containing 1300 bp of the 50-flanking region of the mouse MMP-9 gene has been previously described [20]. Mouse myogenin promoter (G133-luc) was kindly provided by Dr. Zhenguo Wu (Hong Kong University of Science & Technology, Hong Kong, China). G133-Luc and its derivatives were generated by inserting the XbaI-BglII fragments from the corresponding chloramphenicol acetyltransferase constructs into the HindIII-BglII-digested pXP2, respectively [24]. ERK1/2 signaling was determined using pSRE-Luc (kindly provided by Dr. A. Corbi, CIB, Madrid, Spain), which contains two copies of the c-fos SRE (nucleotides −357 to −275, containing both an SRF binding site and an adjacent Ets motif) upstream of a minimal Tk promoter and the luciferase gene. PCMV-*β*-galactosidase expression vector was kindly provided by Dr. C. Bernabeu (CIB, Spain) and was used as a positive control for transfection efficiency. Constitutively active and dominant-negative MEK1-HA tagged constructs were kindly provided by Dr. Jacques Pouyssegur (University of Nice-Sophia Antipolis, France). The constitutively active mutant of MEK1 (CA-MEK1) was designed by substitution of the regulatory phosphorylation sites, Ser218 and Ser222, with aspartic acid (S218D/S222D mutant) as described previously [25]. The pcDNA3.1 empty vector was from Invitrogen.

C2C12 myoblasts seeded in 24-well plates (2 × 10^5^ cells/well) were transfected with different plasmids using Turbofect transfection reagent according to the manufacturer's protocol (Fermentas, St. Leon-Rot, Germany). After 24 hours of treatment, cells were lysed with 50 *μ*L of passive lysis reagent and firefly luciferase activity (Promega, Madison, WI, USA) was determined. *β*-galactosidase activity (Tropix, Bedford, MA, USA) was measured as an internal control for transfection efficiency.

### 2.7. Statistical Analysis

All experiments were performed at least three times and representative results are shown. Data are given as means ± SEM. Statistical significance was evaluated using one-way ANOVA followed by Tukey's test between individual groups. Differences were considered significant at a value of *p* < 0.05 (*∗*) and *p* < 0.001 (*∗∗*) between control samples and treated samples. Differences were considered significant at values *p* < 0.001 (##) between IL-17-treated samples and Doxycycline plus IL-17-treated samples.

## 3. Results

### 3.1. IL-17 Increases MMP-9 Production in Myoblast Cells

**Figure 1:**
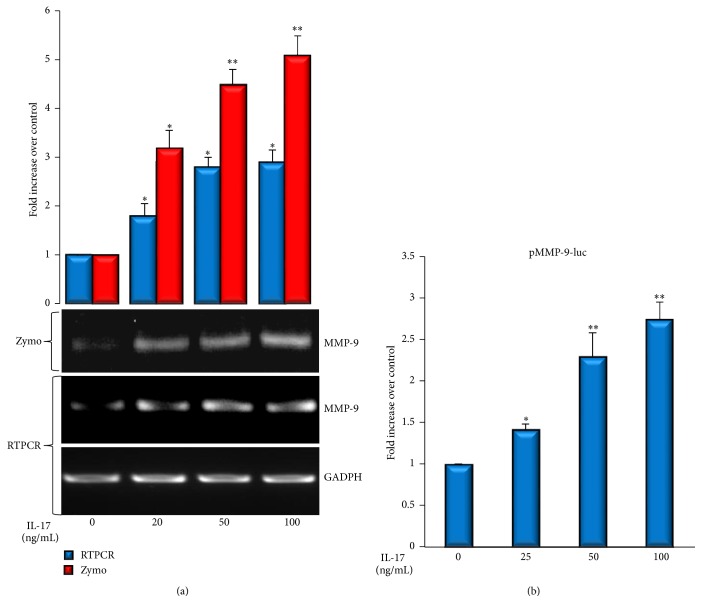
IL-17 enhances MMP-9 production in C2C12 myoblast cells. (a) C2C12 cells were treated with increasing concentrations of IL-17 for 24 h. MMP-9 activity in conditioned media was determined by zymography and MMP-9 expression was determined by RT-PCR analysis. GAPDH was used as a gel loading control. MMP-9 activity is observed in form of clear bands in the gel. Quantification plot is presented above the zymography. (b) MMP-9 promoter transactivity in C2C12 cells transiently transfected with mouse pMMP-9-luc reporter plasmid and treated with increasing amounts of IL-17 for 24 h. Significant difference from the control (cells not treated with IL-17) by *t*-test: ^*∗*^ *p* < 0.05 and ^*∗∗*^ *p* < 0.001.

Firstly, we examined whether IL-17 can regulate the expression and production of MMP-9, a major extracellular protease that can degrade several components of basement membrane and intramuscular connective tissue [26], in cultured C2C12 myoblasts. C2C12 cells were incubated in serum-free medium with increasing amounts of IL-17 for 24 hours and the production of MMP-9 in culture supernatants was measured by gelatin zymography. The treatment with increasing amounts of IL-17 significantly enhanced the activity of MMP-9 and increased MMP-9 mRNA levels (Figure 1(a)), while it did not produce detectable changes in MMP-2 activity (Supplementary Figure  1(a)). Next, we evaluated the effect of IL-17 on the transactivation of MMP-9 promoter in C2C12 cells. Similar to the results obtained in zymography assay and RT-PCR, IL-17 increased the transactivation of MMP-9-specific promoter in a dose-dependent manner (Figure 1(b)).

### 3.2. MMP-9 Production Is Highly Induced by IL-17 during Inhibition of C2C12 Myogenic Differentiation

**Figure 2:**
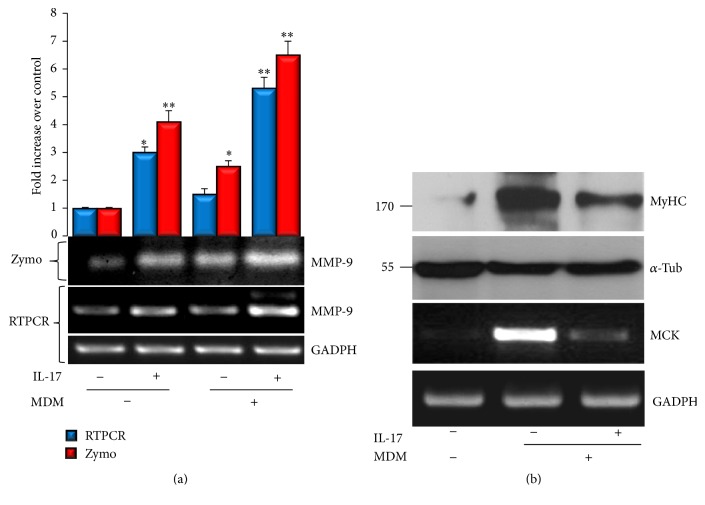
MMP-9 is highly induced by IL-17 during myogenic differentiation of C2C12 cells. (a) C2C12 cells were cultured in myogenic differentiation medium (MDM) with or without 50 ng/mL of IL-17 and compared to cells cultured in GM with or without IL-17. MMP-9 activity in conditioned media was determined by zymography. MMP-9 mRNA transcript production in C2C12 cells was determined by RT-PCR analysis. GAPDH was used as a gel loading control. Quantification plot is presented above the zymography. (b) MCK and MyHC expression in C2C12 cells during myogenic differentiation with or without 50 ng/mL of IL-17 determined by RT-PCR and Western blot analysis, respectively. *α*-tubulin and GAPDH were used as gel loading controls.

To determine whether IL-17 affects MMP-9 expression during myogenic differentiation, C2C12 cells were induced to differentiate under IL-17 treatment and MMP-9 expression was determined after three days. As presented in Figure 2(a), both in GM and under differentiation induction, increased MMP-9 production and expression were noticed in response to IL-17 treatment, as determined by both zymography and RT-PCR. Moreover, during C2C12 differentiation induction, IL-17 inhibited the expression of myogenesis markers myosin heavy chain (MyHC) and muscle creatine kinase (MCK), determined by Western blot and RT-PCR, respectively (Figure 2(b)), suggesting that IL-17 induces MMP-9 expression concomitantly with the inhibition of myogenic differentiation of C2C12 cells. The enhanced MMP-9 and reduced MCK expression paralleled with the myotubes atrophy induced by IL-17, as shown in Supplementary Figure  1(b).

### 3.3. Doxycycline Reverts Myogenesis Inhibition Induced by IL-17

**Figure 3:**
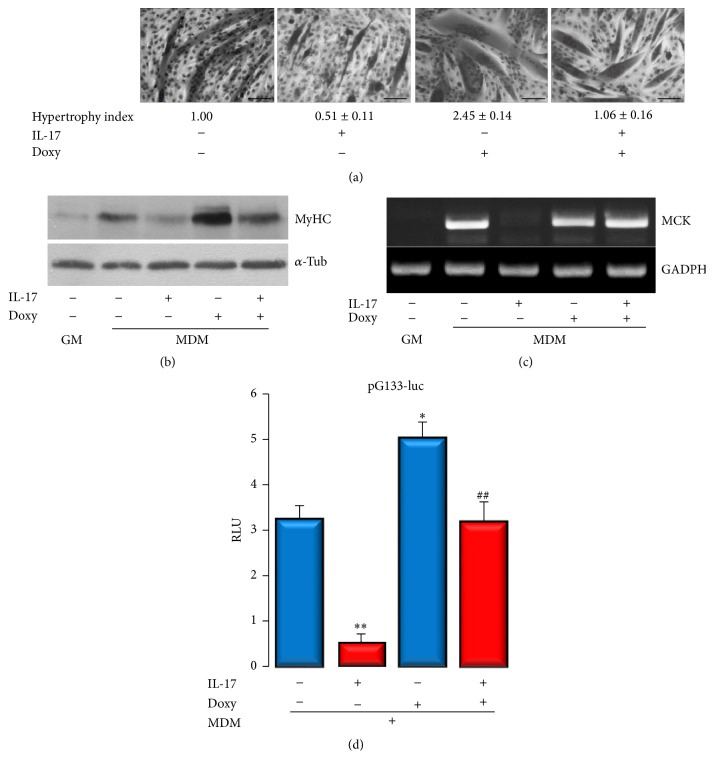
Doxycycline reverts myogenesis inhibition induced by IL-17. (a) Hypertrophy index of myotubes was measured after 4 days of cultivation in differentiation medium with or without Doxy and IL-17. Hypertrophic myotubes are determined by the average myotube diameter. (b) MyHC protein expression under conditions described above determined by Western blot. *α*-tubulin is used as gel loading control. (c) MCK expression under conditions described above determined by RT-PCR. GAPDH is used as gel loading control. (d) Myogenin promoter (pG133-luc) transactivity determined by luciferase assay. RLU: relative luciferase activity.

Modulation of MMP-9 is beneficial for the improvement of skeletal muscle regeneration in mdx mice [12, 16]. It has been demonstrated that the MMP inhibitor Doxy decreases the level of MMP-9 in biceps brachii and diaphragm of the mdx mice [15]. Next, we aimed to elucidate whether MMP-9 modulated by Doxy may protect C2C12 cells from the inhibition of myogenesis induced by IL-17. The optimal concentration for Doxy was set to 10 *μ*g/mL after dose-dependent experiment (data not shown). Doxy (10 *μ*g/mL) improved myogenesis (Figure 3(a)) by producing more hypertrophic myotubes, determined by the average myotube diameter (2.45-fold compared with cells grown in MDM only). In addition, Doxy protected myotubes formation in the presence of IL-17, as the cells had the hypertrophy index similar to that in control. Interestingly, myotubes appeared to be shorter in the presence of IL-17 than myotubes formed without IL-17 treatment. The effect of Doxy was confirmed by muscle-specific protein MyHC expression. As shown in Figure 3(b), MyHC was highly expressed in the presence of Doxy during myogenesis induction, while, in the presence of IL-17, C2C12 cells expressed MyHC at similar levels as cells cultivated in MDM only. Moreover, IL-17-induced repression of the MCK gene expression (Figure 3(c)) was completely conversed by Doxy as well as the IL-17-induced repression of myogenin promoter (pG133-luc) transactivity (Figure 3(d)).

### 3.4. Doxycycline Inhibits MMP-9 Induced by IL-17

**Figure 4:**
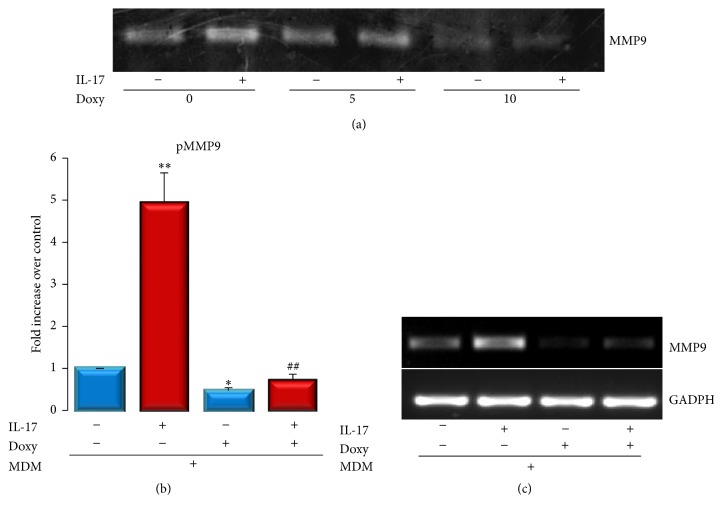
IL-17-stimulated MMP-9 expression is inhibited by Doxycycline. (a) IL-17 induced MMP-9 activation in the presence or absence of 5 or 10 *μ*g/mL of Doxy determined by zymography. (b) Promoter transactivation induced by IL-17 in the presence or absence of Doxy. (c) IL-17-induced MMP-9 mRNA expression determined by RT-PCR. GAPDH is used as gel loading control.

Beyond its capacity to inhibit MMP activity, Doxy has been demonstrated to inhibit MMP-9 protein expression [27]. Next, we determined whether Doxy can transcriptionally regulate IL-17-induced MMP-9 expression in C2C12 cells. As shown in Figure 4(a), Doxy (5 or 10 *μ*g/mL) inhibited both basal MMP-9 expression levels and the capacity of IL-17 to increase the secreted activity of MMP-9. This inhibition seems to be due to transcriptional regulation, since Doxy also reduced both promoter transactivation and MMP-9 mRNA expression induced by IL-17 (Figures 4(b) and 4(c)). Moreover, similar Doxy concentrations did not significantly modify MMP-2 secreted activity (Supplementary Figure  1(c)).

### 3.5. Doxycycline Inhibits ERK1/2 MAPK Activation Induced by IL-17

**Figure 5:**
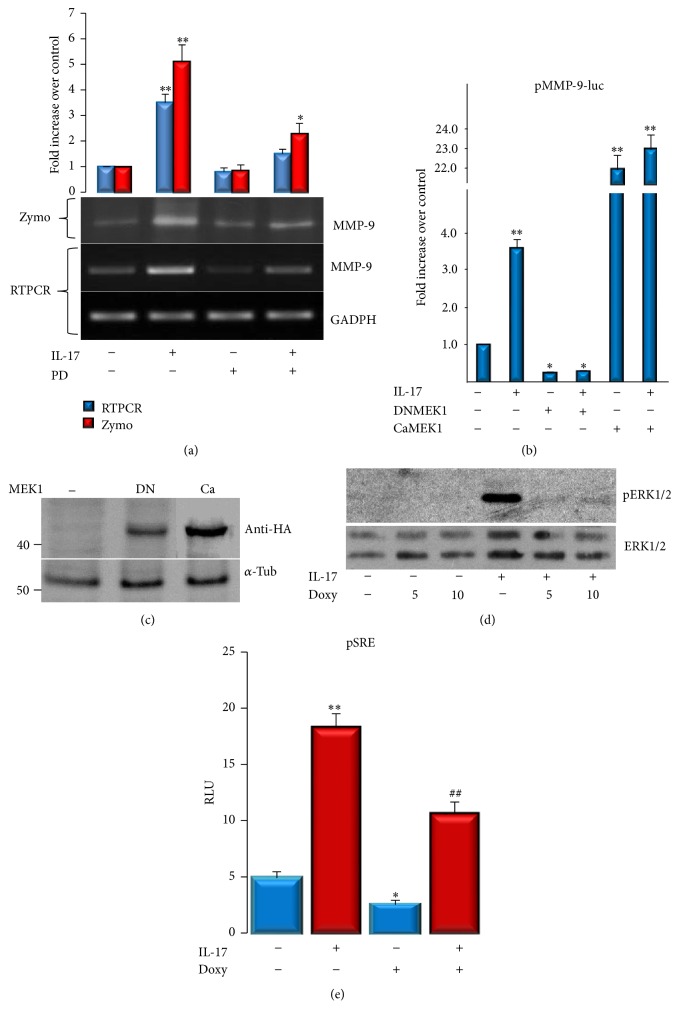
Doxycycline reduces MMP-9 expression by inhibiting IL-17-stimulated ERK1/2 activation in C2C12 cells. (a) MMP-9 expression in C2C12 cells determined by zymography and RT-PCR. Cells were, in the presence or absence of PD98059 (25 *μ*M), treated with or without 50 ng/mL IL-17 for 24 h. GADPH was used as a gel loading control. Numbers represent average densitometry values over control with value 1. (b) Cells were transiently transfected with mouse MMP-9 promoter and cotransfected with DNMEK1 or CaMEK1 mutants or cotreated with PD98059 (25 *μ*M). After the cells were treated for 24 h without or with IL-17 (50 ng/mL), luciferase activities were determined. Results are presented over control with value 1. Significant differences from the control (cells not treated with IL-17) by *t*-test: ^*∗*^ *p* < 0.05 and ^*∗∗*^ *p* < 0.001. (c) Expression of dominant-negative (DN) and constitutively active (Ca) mutants of MEK1 transiently transfected C2C12 cells was confirmed by their reactivity to anti-HA antibody (d) IL-17-induced ERK1/2 phosphorylation in the presence or absence of Doxy (5 or 10 *μ*g/mL) was determined by Western blot. (e) Transactivation of pSRE-luc reporter by IL-17 in the presence or absence of Doxy.

Subsequent experiments addressed the involvement of intracellular signaling pathways in IL-17-induced MMP-9 expression in C2C12 cells. We have previously shown that IL-17 activates ERK1/2 in C2C12 cells [19], and, in order to clarify whether ERK1/2 pathway mediates IL-17-induced MMP-9 production and expression, C2C12 cells were treated with IL-17 in the presence of MEK1,2 inhibitor, PD98059, and the MMP-9 protein and mRNA expression were determined. As shown in Figure 5(a), cotreatment of the cells with IL-17 and MEK1,2 inhibitor significantly blocked the increment of both MMP-9 activity and mRNA expression induced by IL-17.

Furthermore, the IL-17-increased MMP-9-specific promoter transactivation was dramatically inhibited by dominant-negative (DN) MEK1 promoter, while the cotransfection with constitutively active MEK1, independently of IL-17, significantly transactivated the MMP-9 promoter (Figure 5(b)). ERK1/2 signaling appeared to be necessary and sufficient to mediate IL-17-induced MMP-9 expression in C2C12 cells. The appropriate expression of both dominant-negative and constitutively active (Ca) mutants of MEK1 in transiently transfected C2C12 cells was confirmed by their reactivity to anti-HA antibody (Figure 5(c)). It has also been shown that, independently of its capacity to inhibit MMP activity, Doxy inhibits MAPK activation [27]. Since ERK1/2 signaling seems to be critical for IL-17's induction of MMP-9, we determined whether Doxy affects the activation of this pathway by IL-17. As shown in Figure 5(d), the capacity of IL-17 to induce ERK1/2 phosphorylation was dramatically inhibited in the presence of Doxy (5 or 10 *μ*g/mL). This result paralleled with the transactivation capacity of this signal, since Doxy inhibited the capacity of IL-17 to induce the transactivation of pSRE-luc reporter (Figure 5(e)), thus indicating that Doxy inhibits IL-17-induced MMP-9 by downregulating ERK1/2 signal transduction.

## 4. Discussion

Skeletal muscle repair is a highly synchronized process involving various cellular and molecular responses. Coordination between inflammation and regeneration is believed to be crucial for the beneficial outcome of the repair process following muscle damage [28, 29]. During this process, the extracellular matrix surrounding skeletal muscle cells has an important role in maintaining the structure of the muscle, acting as a scaffold for myofiber regeneration [10, 30]. Therefore, remodeling of ECM is important for muscle maintenance and repair in normal and pathological conditions. Matrix metalloproteinases are key players in skeletal muscle ECM degradation and regeneration [10]. In addition to high levels of IL-17, elevated levels of MMP-9 have also been observed in skeletal muscle during inflammatory myopathies [10]. However, regulation of MMP-9 expression by IL-17 in muscle cells has not been elucidated yet.

In the present work, our results demonstrated that IL-17 increases the expression and production of MMP-9 in mouse myoblast cells C2C12, while not significantly affecting the constitutively expressed MMP-2 (Figure 1 and Supplementary Figure). Expression of MMP-2 and MMP-9 has been previously reported in myogenic cells of various species with a general consensus about the constitutive expression of MMP-2 by muscle cells and more controversial MMP-9 expression [31]. Our results obtained using C2C12 cells showed that IL-17 increases MMP-9 expression at both transcriptional and protein level. Since IL-17 is primarily a proinflammatory cytokine, this result is in agreement with previous observations that MMP-9 expression is related to the inflammatory response [10], as well as that MMP-9 upregulation in muscle tissue appears to be a common finding in all inflammatory myopathies [31]. In addition, it is consistent with the abundant evidence, indicating that MMP-9 gene expression is, to a large extent, tightly regulated at transcriptional level [32]. Furthermore, it is known that proinflammatory cytokines are able to activate muscle satellite cells, possibly by inducing MMP-9 expression [10].

During myogenic differentiation, basal MMP-9 expression was increased and its expression was higher in the presence of IL-17. This effect was accompanied with the capacity of IL-17 to inhibit the expression of myogenic differentiation marker MCK, demonstrating that IL-17 enhances MMP-9 expression concomitantly with the inhibition of myogenic differentiation of C2C12 cells. It could be speculated that chronic exposure to IL-17, by inducing MMP-9 expression, may affect the homeostasis of muscle cells, as well as their ECM remodeling, which could then contribute to the development of inflammatory muscle diseases. In pathological conditions, abnormal increment in MMP-9 expression and activity may produce excessive degradation of type IV collagen in the skeletal muscle basement membrane, which in cooperation with other MMPs can lead to skeletal muscle tissue loss [10, 33].

Doxycycline is the most potent MMP inhibitor among the Food & Drug Administration USA/Health Canada-approved tetracyclines, which inhibits MMP activity at plasma levels lower than those needed for its antimicrobial effect [34, 35]. In addition, Doxy, beyond its role in proteolysis, possesses biological roles in inflammation, angiogenesis, apoptosis, metal chelation, ionophoresis, and bone metabolism [36]. Our results demonstrated that Doxy may rescue* in vitro* myoblast differentiation by reverting IL-17 inhibition of myogenesis and MMP-9 induction. Moreover, during the induction of myoblastic differentiation, Doxy appeared to increase myotube mass, indicated by the hypertrophic index and MyHC expression. Interestingly, although MMP-9 inhibition was shown to improve soleus muscle regeneration* in vivo*, it has been reported by Zimowska et al. that Doxy may delay C2C12 differentiation* in vitro* [16]. In the aforementioned study, authors used Doxy at concentration of 60 *μ*M which is approximately three times higher than the highest concentration utilized in this study (10 *μ*g/mL or 22 *μ*M). We observed that 5 *μ*g/mL (11 *μ*M) is enough to induce myotube hypertrophy. This may indicate that the Doxy concentration is important to achieve the desired effects, and higher concentration may impair myogenesis by excessive inhibition of MMP-9, which is also necessary for normal myogenic differentiation where MMP-9 is mainly secreted during prefusion stages [10]. However, during inflammatory process, increased MMP-9 secretion may disturb the proteolytic homeostasis, thus impairing muscle cell differentiation, as observed here for IL-17.

As mentioned above, Doxycycline may also affect different biological processes which can be explained by its capacity to influence the activation of intracellular signals such as ERK1/2 signaling [27, 37]. In this line, IL-17 was shown to trigger numerous signal transduction pathways, including ERK1/2 and p38 MAPKs [4]. In accordance with our previous work [19], we confirmed here that IL-17 is able to enhance phosphorylation of ERK1/2 in C2C12 cells, and we also showed that this pathway was essential for the induction of MMP-9 expression by IL-17. Our results indicate that Doxycycline strongly inhibits IL-17-induced MMP-9 expression and ERK1/2 activation. Furthermore, we demonstrated that MEK1,2-ERK1/2 inhibition by using the chemical inhibitor PD98059 reverted the suppressive effect of IL-17 on C2C12 myogenic differentiation [19]. Moreover, PD98059 was shown to enhance C2 myoblast cell differentiation* in vitro* [38], thus suggesting that Doxy may rescue C2C12 myogenic differentiation by downregulating IL-17 activation of ERK1/2 signaling which is necessary for the increased MMP-9 expression.

Interestingly, IL-17 did not affect the capacity of C2C12 cells to secrete MMP-2, and similar result was obtained by treating cells with Doxy (Supplementary Figure), indicating that MMP-2 is not a target gene neither for IL-17 nor for Doxy. It has been demonstrated that during C2C12 differentiation MMP-2 expression is mainly unaltered, since MMP-2 is expressed in these cells by a constitutive mechanism [39, 40]. Although, in some inflammatory myopathies, such as polymyositis, dermatomyositis, and inclusion body myositis, both MMP-2 and MMP-9 are upregulated, their balance depends on the type of disease and local presence of various factors. In this regard, upregulated MMP-9 in muscle tissue appears to be a common finding in all inflammatory myopathies, while MMP-2 seems to be affected to a lesser extent [31]. Results presented here, together with our previously published results, which demonstrated that IL-17 does not affect MMP-2 expression, while it inhibits the expression of uPA in C2C12 cells [41], further confirm the complex role that IL-17 has on proteolytic enzymes expression and activity in myoblasts. This suggested that MMP-9/MMP-2 disbalance may be one of the major characteristics of myopathies. Therefore, IL-17 may affect C2C12 myogenic differentiation by increasing MMP-9 expression, while Doxy can protect and preserve MMP-9/MMP-2 balance during myogenesis via ERK1/2 by reducing MMP-9 expression. Of note, it would be of interest to explore the relative ratio between MMPs and tissue inhibitors of MMPs (TIPMs), as there is no available data about this mode of regulation during muscle inflammation and regeneration involving IL-17, although there are some reports about their mutual regulation during muscle differentiation [40].

Finally, in Duchenne muscular dystrophy (DMD), primary deficiency of dystrophin leads to several secondary pathological changes including ECM breakdown, inflammation, and fibrosis, all of which require MMP activity. Besides revealing that MMP-9 inhibition augments the proliferation of satellite cells and changes the immune cell milieu as well as expression of significant ligands, receptors, and signaling pathways, this group showed that inhibition of MMP-9 dramatically improves the engraftment of transplanted myoblasts in skeletal muscle of mdx mice (a mouse model of DMD) [42]. Together with our results, this finding adds value to the importance of MMP-9 in muscle regeneration and suggests this enzyme as a possible target in muscle regeneration and repair.

However, the effect of IL-17 in the regulation of MMP-9 production in myoblast cells and its association with physiological events and/or pathological conditions as well as Doxycycline inhibition of IL-17-induced MMP-9 expression should be additionally confirmed in further* in vivo* studies, as this will help determine its role in muscle disease and regeneration.

## 5. Conclusions

Our data provide evidence that Doxycycline inhibits the capacity of IL-17 to induce MMP-9 expression in myoblast cells by regulating the activation of ERK1/2 parallel with its ability to protect C2C12 myoblast cells from the inhibitory effect that IL-17 has on myogenic differentiation. The regulation of IL-17–induced MMP-9 expression by Doxycycline may be useful to control the proteolytic status during muscle regeneration and muscle inflammatory diseases.

## Supplementary Material

Supplementary Figure:
(A) MMP-2 activity in C2C12 cells treated with increasing concentrations of IL-17 determined by zymography.
(B) Myotube formation in C2C12 cells grown in GM and MDM with or without IL-17 (50 ng/ml).
(C) MMP-2 activity in C2C12 cells treated with increasing concentrations of Doxy determined by zymography.
Supplementary table: primer sequences and PCR settings.
