# Supplementary material for: Doxycycline Inhibits IL-17-Stimulated MMP-9 Expression by Downregulating ERK1/2 Activation: Implications in Myogenic Differentiation
Source: Mediators Inflamm. 2016 Nov 30;2016:2939658. doi: 10.1155/2016/2939658 (PMC5155103; doi:10.1155/2016/2939658)
Supplement: Supplementary file 1 — Supplementary Figure: (A) MMP-2 activity in C2C12 cells treated with increasing concentrations of IL-17 determined by zymography. (B) Myotube formation in C2C12 cells grown in GM and MDM with or without IL-17 (50 ng/ml). (C) MMP-2 activity in C2C12 cells treated with increasing concentrations of Doxy determined by zymography. Supplementary table: primer sequences and PCR settings. [file 2939658.f1.pdf]

(A)

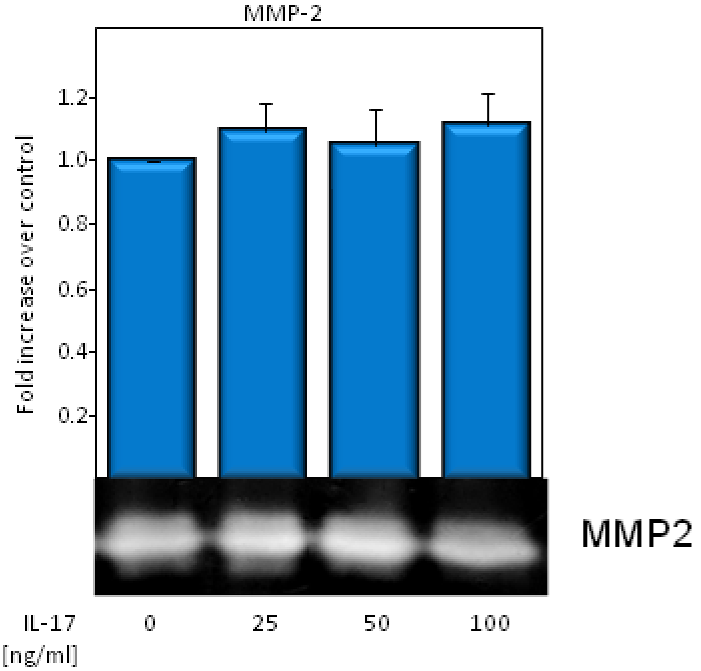

(B)

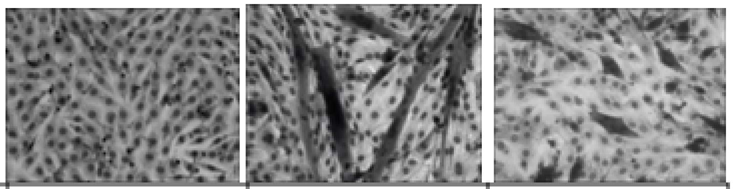

IL-17	-	-	+
MDM	-	+	+

(C)

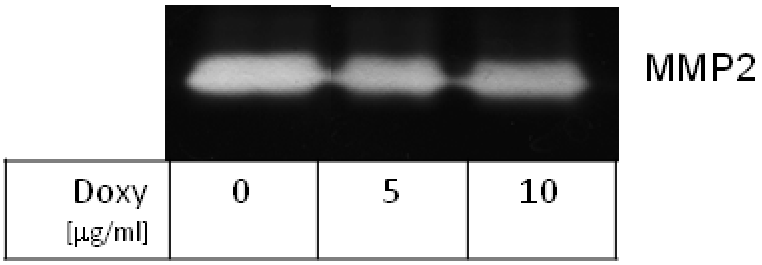

Doxy [μg/ml]	0	5	10

Supplementary Figure

Gene	Primer sequence	Size (bp)	Temp (^{o}C)	Number of cycles	Ref.
**MMP9**	Foward: 5'-ACC-ACC-ACA-ACT-GAA-CCACA-3'<br>Reverse: 5'-ACC-AAC-CGT-CCT-TGA-AGA-AA-3'	304	50	28	(1)
**MMP-2**	Forward :5'-GGCTGGAACACTCTCAGGAC-3'<br>Reverse: 5'-CGATGCCATCAAAGACAATG-3'	213	50	28	(2)
**MCK**	Forward: 5'-CAA-TAA-GCT-TCG-CGA-TAA-GGA-G-3'<br>Reverse: 5'- AGG-AAG-CTT-TTG-TTG-TCG-TTG-3'	561	48	30	(3)
**GADPH**	Forward: 5'-ACCACAGTCCATGCCATCAC-3'<br>Reverse: 5'-TCCACCACCCTGTTGCTGTA-3'	452	52	26	(1)

Supplementary table 1: primer sequences and PCR settings.

References

(1) J.F. Santibanez, J. Kocić, A. Fabra, A. Cano, M. Quintanilla, ''Rac1 modulates TGF-beta1-mediated epithelial cell plasticity and MMP9 production in transformed keratinocytes''. *FEBS Lett* vol 584, no. 11, pp. 2305-2310, 2010.

(2) Kurata K, Maruyama S, Kato S, Sato W, Yamamoto J, Ozaki T, Nitta A, Nabeshima T, Morita Y, Mizuno M, Ito Y, Yuzawa Y, Matsuo S. Tissue-type plasminogen activator deficiency attenuates peritoneal fibrosis in mice. Am J Physiol Renal Physiol 2009;297(6):F1510-7.

(3) Y. Gutierrez-Martin, F.J. Martin-Romero, F. Henao, ''Store-operated calcium entry in differentiated C2C12 skeletal muscle cells''. *Biochim. Biophys. Acta,* vol 1711, no.1, pp. 33-40, 2005.
